# Novel Iron-Chelator DIBI Inhibits *Staphylococcus aureus* Growth, Suppresses Experimental MRSA Infection in Mice and Enhances the Activities of Diverse Antibiotics *in vitro*

**DOI:** 10.3389/fmicb.2018.01811

**Published:** 2018-08-14

**Authors:** Maria del Carmen Parquet, Kimberley A. Savage, David S. Allan, Ross J. Davidson, Bruce E. Holbein

**Affiliations:** ^1^Chelation Partners Inc., Halifax, NS, Canada; ^2^Department of Microbiology & Immunology, Dalhousie University, Halifax, NS, Canada; ^3^Queen Elizabeth II Health Sciences Centre, Nova Scotia Health Authority, Halifax, NS, Canada

**Keywords:** iron chelator, *Staphylococcus aureus*, DIBI, antibiotic, hydroxypyridinone, deferiprone

## Abstract

DIBI, a purpose-designed hydroxypyridinone-containing iron-chelating antimicrobial polymer was studied for its anti-staphylococcal activities *in vitro* in comparison to deferiprone, the chemically related, small molecule hydroxypyridinone chelator. The sensitivities of 18 clinical isolates of *Staphylococcus aureus* from human, canine and bovine infections were determined. DIBI was strongly inhibitory to all isolates, displaying approximately 100-fold more inhibitory activity than deferiprone when compared on their molar iron-binding capacities. Sensitivity to DIBI was similar for both antibiotic-resistant and -sensitive isolates, including hospital- and community-acquired (United States 300) MRSA. DIBI inhibition was primarily bacteriostatic in nature at low concentration and was reversible by addition of Fe. DIBI also exhibited *in vivo* anti-infective activity in two distinct MRSA ATCC43300 infection and colonization models in mice. In a superficial skin wound infection model, topical application of DIBI provided a dose-dependent suppression of infection along with reduced wound inflammation. Intranasal DIBI reduced staphylococcal burden by >2 log in a MRSA nares carriage model. DIBI was also examined for its influence on antibiotic activities with a reference isolate ATCC6538, typically utilized to assess new antimicrobials. Sub-bacteriostatic concentrations of DIBI resulted in Fe-restricted growth and this physiological condition displayed increased sensitivity to GEN, CIP, and VAN. DIBI did not impair antibiotic activity but rather it enhanced overall killing. Importantly, recovery growth of survivors that typically followed an initial sub-MIC antibiotic killing phase was substantially suppressed by DIBI for each of the antibiotics examined. DIBI has promise for restricting staphylococcal infection on its own, regardless of the isolate’s animal source or antibiotic resistance profile. DIBI also has potential for use in combination with various classes of currently available antibiotics to improve their responses.

## Introduction

*Staphylococcus aureus* is a normal colonizer of the skin and mucosal surfaces (e.g., nares) of around 30% of healthy humans but this opportunistic pathogen also causes a range of serious wound, lung and bloodstream infections (Liu, 2009; Tong et al., 2015). Staphylococci are also prevalent in animal infections. For example, up to 80% of clinical and subclinical cases of bovine mastitis involve *S. aureus* resulting in worldwide economic losses of US $35B/year (Hamid et al., 2017). The staphylococci are also prevalent in various infections of companion animals such as with canine otitis where it along with closely related *S. pseudintermedius* have been found in >36% of all cases (De Martino et al., 2016). The growing spread of methicillin resistant *S. aureus* (MRSA) among humans (Tong et al., 2015; Ventola, 2015) and from animals in close contact with humans such as dogs (De Martino et al., 2016) dairy cattle or pigs (Vincze et al., 2014; Hamid et al., 2017) along with the significant mortality (14%) seen with invasive human MRSA infection has led to the designation of MRSA as a serious health threat (Frieden, 2013). The prospect for developing an effective staphylococcal vaccine appears to remain poor, possibly due in part to the wide range of infections involved with this microorganism (Proctor, 2012).

Given these various factors, our dependence on antibiotic therapy for staphylococcal infections of man and animals will likely continue. Thus, there is a need for new treatment approaches to deal with the MRSA threat (Frieden, 2013; Ventola, 2015). New anti-infectives that might work on their own or also in combination with antibiotics to improve efficacy, represent a new approach. In this regard, agents that target microbial iron acquisition have been recognized for their potential (Ballouche et al., 2009; Luo et al., 2014). Microbial pathogens have broad-based and irreplaceable needs for essential iron and they must obtain sufficient iron supplies from their host during infection. A key aspect of the vertebrate host’s innate defense relates to restricting availability of iron to microbes during infection (Weinberg, 2009). The two interrelated aspects of microbial iron dependence and host iron limitation during infection provide good foundations for the potential use of iron sequestering anti-infective agents that might interfere with microbial iron acquisition and/or augment host iron restriction mechanisms.

*Staphylococcus aureus* is very adept with iron acquisition being able to access host transferrin, lactoferrin and heme iron sources, produces its own siderophores and is also able to access xenosiderophores including deferoxamine (Hammer and Skaar, 2011 and Haley and Skaar, 2012). The recent demonstration that during experimental infection there is upregulation of the *S. aureus* FhuD2 iron acquisition receptor which binds hydroxamate xenosiderophores such as deferoxamine (Bacconi et al., 2017) is significant. Additionally, various other siderophore Fe acquisition genes are upregulated in *S. aureus* while colonizing the nares (Chaves-Moreno et al., 2016). These various findings provide strong evidence for both active host iron restriction and bacterial iron acquisition response during staphylococcal infection.

Deferoxamine (Desferal^®^ Novartis) is used clinically to treat transfusional iron overload and its use has been associated with increased incidence of staphylococcal infection due to the ability of this bacterium to directly access various hydroxamate siderophores including deferoxamine using its FhuD1 and FhuD2 iron acquisition systems (Hammer and Skaar, 2011; Cassat and Skaar, 2012). Deferiprone is another clinically used iron chelator that has been investigated as a potential anti-infective but it has shown relatively poor anti-staphylococcal activity with MICs typically >68 μg/ml, i.e., >0.5 mM (Huber et al., 2007; Kim and Shin, 2009; Thompson et al., 2012). Deferasirox, yet a third clinically used iron chelator displayed relatively weak activity for *S. aureus* growth inhibition *in vitro* with an MIC of 50 μg/ml, i.e., 0.13 mM (Luo et al., 2014). The relatively high concentrations of these hematologically useful iron chelators required to inhibit microbial growth, their potential use by *S. aureus* or other pathogens as iron sources, especially deferoxamine (Thompson et al., 2012) and potential issues as to their toxicity, especially deferasirox (Kontoghiorghes, 2011) have severely limited their use as prospective anti-infectives.

We hypothesized that the relatively weak antimicrobial activities of small molecule chelators such as deferiprone might be improved by incorporating similar chelator-functionality into water soluble polymers of a sufficiently larger molecular size, i.e., >1500 Da such that these compositions would still sequester iron but would be less accessible by microbes for their bound iron. This approach has led to a family of potential anti-microbial chelating compositions (Holbein et al., 2007, 2011; Holbein and Mira de Orduña, 2010; Ang et al., 2018). The antimicrobial activities and chemical properties of one such hydroxypyridinone composition, DIBI, have been reported previously (Holbein and Mira de Orduña, 2010; Ang et al., 2018; Savage et al., 2018).

Here, we report the activity of DIBI in comparison to deferiprone, its small molecule (139 Da) chemical relative, against diverse *S. aureus* clinical isolates from human, cattle and canine infections. The *in vivo* activity of DIBI against a MRSA isolate and the enhancing activity of DIBI in combination with gentamicin, ciprofloxacin, and vancomycin are also reported.

## Materials and Methods

### Bacterial Strains and Cultivation

Bacterial isolates used in this study are listed in **Table 2**. Strain 38 was obtained from Mark Wilcox, University of New South Wales Australia and WBG525 was obtained from Warren Grubb, Curtin University, Australia. Isolates 12-334-07086 and 14-268-06583 were obtained from the Capital District Health Authority, Halifax, NS, Canada. The canine otitis isolates were obtained from Luisa De Martino, University of Naples, Italy. The bovine mastitis isolates were obtained from the Canadian Mastitis Culture Collection, Montreal, Canada. All isolates were routinely cultured from frozen stocks (-80°C) and maintained on Trypticase Soy Agar (TSA, Sigma-Aldrich) or Blood Agar (BA, Becton Dickinson). Liquid cultures were grown in Mueller-Hinton Broth MHB (Oxoid) or Roswell Park Memorial Institute Medium (RPMI 1640, Sigma-Aldrich) supplemented with 2% (w/v) glucose, buffered with 0.165M 3-(*N*-morpholino)-propanesulfonic acid. Partially deferrated MHB and RPMI, extracted with FEC1 to remove excess Fe (MHB-FEC1 and RPMI-FEC1) were prepared using FEC1 as previously described (Holbein and Mira de Orduña, 2010). Cultures were grown at 35–37°C with shaking. All experiments were executed following approved biosafety and biosecurity standards (Public Health Agency of Canada) in a level 2 biosafety laboratory.

### Iron-Chelators, Antimicrobials and Iron Supplementation

DIBI and FEC1 were supplied by Chelation Partners Inc. Gentamicin (GEN), ciprofloxacin (CIP), and vancomycin (VAN) (Sigma-Aldrich) were prepared as 10 mg/mL stocks in water while deferiprone (DFP) from Sigma-Aldrich was dissolved in RPMI. DIBI stocks were prepared in either water (10 mg/mL) or RPMI (200 mg/mL). Exogenous iron was added to growth media as ferric citrate (Sigma-Aldrich) in RPMI. All stock solutions were filter-sterilized (0.2 μm filter) before use.

### Iron Analyses

For determination of trace elements, 10 mL samples were stabilized with 1.0% (final) ultra-high purity nitric acid and stored in acid-cleaned polycarbonate centrifuge tubes. Treated samples were kept unfrozen until analyzed to avoid the formation of iron colloids that are difficult to dissolve. The samples were number-coded prior to submission to the Trace Element Research Group, Wisconsin State Laboratory of Hygiene, University of Wisconsin-Madison, which performed analysis of 50 elements using a magnetic sector inductively coupled plasma mass spectrometry ICP-MS.

### Susceptibility Testing

We grew cultures overnight in MHB-FEC1 and then tested MIC susceptibilities to DIBI, DFP, GEN, CIP and VAN in RPMI using the broth microdilution method in 96-well round-bottomed plates. MHB-FEC1 cultures were diluted to an optical density (OD600 nm) of 0.1 and MIC plates were inoculated into RPMI at a final dilution of 1/200 (approximately 1–5 × 10^5^ CFU/mL). Chelator and antibiotic stocks were diluted in RPMI with serial 1/2 dilutions made. Negative and positive controls were tested in parallel. MIC plates were incubated at 35°C for 48 h and the MIC value was defined as the lowest concentration of chelator or antibiotic required to inhibit visible growth at 24 h incubation. Results were also read after 48 h incubation to examine for relative duration of growth inhibition. At least two independent experiments with 2–4 replicates in each were performed.

### Effect of DIBI and Media Iron Concentration on Bacterial Growth

DIBI was added at concentrations ranging from 0.01 μM (0.09 μg/mL) to 1 μM (9 μg/mL) into RPMI or RPMI-FEC1 to assess the effect of iron sequestration on growth. Ferric citrate addition was used to assess reversal on growth inhibition in RPMI-FEC1 and to cultures treated with DIBI. Cultures were incubated at 35–37**°**C with shaking and OD (600 nm) readings were taken over a 24 h incubation period for replicate experiments.

### Wound Infection Testing

Infection testing with BALB/c female mice using the superficial skin wound infection model first described by Kugelberg et al. (2005) with *S. aureus* ATCC43300 was performed by an external clinical research organization, Jubilant Drug Discovery and Development Services Inc. (Kirkland, QC, Canada). All animal experiments were first approved by the Institutional Ethics Committee of Jubilant Biosys Ltd., and were conducted in full accordance with the CPCSEA (India) guidelines for the use of experimental animals. Mice, 9 week old, nulliparous and non-pregnant were acclimatized for 5 days prior to use and confirmed to be healthy and free of clinical signs before use. DIBI was formulated in a cream-based vehicle at three different concentrations, 0.5, 1, and 2% (w/w). A preliminary vehicle-only sham treatment was done to both validate the model and ensure the vehicle had no effects on its own. Test DIBI creams were supplied to the animal testing facility as number-coded materials.

*Staphylococcus aureus* ATCC43300 was cultured in TSB overnight at 37°C with shaking. Bacteria were harvested by centrifugation, washed once, re-suspended in PBS, and OD600 nm measurement was used to adjust the inoculum to approximately 10^11^ CFU/mL. Actual CFU of inocula were determined by plate counting. On the day of wound creation, mice were anesthetized intraperitoneally with ketamine –xylazine cocktail (100 mg/kg and 20 mg/kg, respectively), fur was shaved from a 2 cm^2^ dorsal area and the exposed skin was stripped (7–10 successive times) using a fresh elastic adhesive bandage (3M) for each stripping. After stripping, the skin became visibly damaged and was characterized by reddening and glistening but typically there was no bleeding. Skin infection was created by applying 10 μL of bacterial cell suspension containing approximately 10^8^–10^9^ CFU onto the central area of the stripped skin wound. Uninfected control animals were prepared similarly but received no inoculum. Treatment groups received 100 mg of the appropriate test cream as applied to the central area of wound at 4 and 12 h post infection (PI) and then at each 12 h interval thereafter except there was no treatment applied within 12 h of the conclusion of the testing at day 4. Application of the 0.5, 1, and 2% DIBI creams represented dosages of 20, 40, and 80 mg/kg, respectively. All treatments were administered in a blind fashion. Skin wounds were examined prior to infection, daily and at 4d PI necropsy and were scored at 4d PI as to degree (0–4) of inflammation: 0 = no erythema; 1 = slight erythema; 2 = well-defined erythema; 3 = moderate to severe erythema and 4 = severe erythema (beet red) to eschar formation with injury at depth. Mice were euthanized with excess CO_2_ and after examination the entire infected tissue including several mm of clearly visible non-infected margin was excised. For bacterial burden quantification tissues were homogenized in PBS under aseptic conditions. Viable total *S. aureus* as CFU per entire wound were determined by plate counting in duplicate on mannitol salt agar, with incubation for 48 h at 37°C. The central portion of excised tissue samples at day 4 PI from four additional mice representing each of the control wounded but non-infected and infected sham-treated groups were also harvested, fixed, paraffin-embedded, sectioned and stained with either hematoxylin/eosin (H&E) or crystal violet (CV) for histopathological examination.

### Nares Carriage Testing

Infection testing with male BALB/c mice utilized the nares carriage/colonization model first described by Chhibber et al. (2014) with *S. aureus* ATCC43300 and this was also performed by Jubilant Drug Discovery and Development Services Inc. All animal experiments were first approved by the Institutional Ethics Committee of Jubilant Biosys Ltd. and were conducted in full accordance with the CPCSEA (India) guidelines for the use of experimental animals. Bacterial cultures were grown in brain heart infusion (BHI) medium after its extraction with FEC1 (performed as for MHB above) with incubation at 37°C. Overnight cultures were harvested by centrifugation at 5000 rpm, cells were resuspended and washed once with sterile PBS (pH 7.4) and then re-suspended in PBS with adjustment by dilution to approximately 10^8^ CFU/mL. Plate counting to confirm inoculum CFU/mL was done on nutrient agar plates containing 1 μg/mL of GEN with incubation for 24 h at 37°C. On day -2 and day 0 mice were anesthetized by exposure to 3–5% isoflurane in an oxygen flow set at approximately 1 liter per minute and infection was initiated by nasal instillation to mice held in an upright position of 10 μL (10^6^ CFU) of bacterial suspension into each nostril. Gentle mixing of the bacterial inoculum between animals ensured a uniform suspension. Mice treated at day 2 or day 2+ day 3 PI were anesthetized and treated with 10 μL/nostril of PBS (vehicle control) or 10 μL of 12.5% DIBI (w/v) in PBS, representing a DIBI treatment dosage of 100 mg/kg. On day 5 PI, mice were sacrificed by excess CO_2_ in an enclosed chamber, the area around the nose was wiped with 70% alcohol and the entire nose along with the nasal bone structure was excised using sterile scissors and this was homogenized in 1 mL sterile PBS. Plate counts of serial dilutions of the tissue homogenates were made after 24 h growth on nutrient agar and nutrient agar +1 μg/mL GEN at 37°C.

### DIBI Synergy With Antibiotics *in vitro*

The effects of DIBI on antibiotic activity were assessed in standard checkerboard assays and then were examined in more detail using time-kill/growth assays as follows.

*Staphylococcus aureus* ATCC6538 after overnight growth in MHB-FEC1 was inoculated at 1–5 × 10^5^ CFU/mL into RPMI medium (control) and RPMI containing either antibiotic alone, DIBI alone, or the combination of DIBI and antibiotic. A sub-MIC of DIBI that provided reduced growth was first determined. GEN, CIP, and VAN were each tested at approximately ½–1 MIC to ensure an initial killing phase followed by recovery growth. Cultures were incubated 48 h at 37°C during which viable bacteria were enumerated at various time points with serial dilutions in PBS and plating onto TSA or BA. Colony counts were taken following overnight incubation at 35°C. At least two independent experiments were performed for each antibiotic and DIBI pair.

### Data Analysis

Growth curves and time-kill assay curves are reported as means ± SEM. For *in vitro* studies, data were analyzed using Two-way ANOVA with Bonferroni post-test (GraphPad Prism software). The bacterial counts for the *in vivo* results were graphed as the log_10_ of CFU/wound and these as well as the inflammation scores are reported with their median value. For *in vivo* studies, data were analyzed using the Mann–Whitney *U* test, and additional One-way ANOVA and Tukey analyses were performed (GraphPad Prism software). Significant differences were defined as *p* < 0.05.

## Results

### Establishing Low Host-Relevant Iron Availability *in vitro*

Host iron availability is very low during infection (Weinberg, 2009) and therefore we considered Fe supply in the growth medium before comparing iron chelator activities *in vitro*, i.e., to better approximate relevant *in vivo* Fe availability conditions. MHB is a complex animal protein-based standard bacteriological testing medium that is rich in iron (Girardello et al., 2012). This large excess Fe content (>8 μM, see **Table 1**) is at least 100X the reported <0.1 μM minimum Fe requirements for *S. aureus* growth (Lacasse et al., 2008). Therefore, we extracted the excess Fe content from MHB using FEC1 resin as utilized previously (Holbein and Mira de Orduña, 2010). Fe contents as well as those for other trace metals and essential cations in MHB and MHB extracted with FEC1 (MHB-FEC1) were compared along with RPMI and RPMI after FEC1 extraction (RPMI-FEC1) and these are summarized in **Table 1**.

**Table 1:** Major cations and trace metals in normal and FEC1-extracted culture media.

Medium	Major cation mgL^-^^1^				Trace metal μgL^-^^1^ (μM Fe)					
	Na	K	Ca	Mg	Fe	Mn	Cu	Co	Ni	Zn
MHB	2,523	89	5.9	6.2	488 (8.7)	16.5	31.4	0.88	33.1	757.0
MHB-FEC1	2,637	93	6.0	6.0	26.7 (0.46)	0.21	18.3	0.91	39.7	264.4
RPMI	4,493	207	19	9.9	18.7 (0.33)	1.39	13.6	0.18	2.1	35.6
RPMI-FEC1	4,418	190	17	8.8	4.6 (0.08)	0.06	0.59	0.16	1.3	7.0

*Average values for *n* ≥ 4 replicates; SD not shown +/- < 3%*.

A single shake flask contacting with FEC1 followed by its removal by filtration along with its adsorbed metals provided a large Fe removal (95%) from MHB with a residual concentration similar to that found in RPMI (i.e., <0.5 μM). Extraction of MHB or RPMI did not affect its contents of Na, K, Ca, and Mg or substantially alter other trace metals (i.e., Co, Ni) except for Mn (99% and 95% removal, respectively) as shown in **Table 1**. The Fe concentrations in both RPMI and MHB-FEC1 were sufficient for unrestricted growth with both media providing 24 h Ymax (OD600 nm) values >1 (results not shown).

### Sensitivity to DIBI and Deferiprone

The MICs for DIBI and deferiprone for the 18 *S. aureus* isolates tested are shown in **Table 2**. DIBI was strongly inhibitory for all 18 isolates with a narrow overall MIC range of 1–4 μg/mL, i.e., 0.1–0.4 μM DIBI. Significantly, antibiotic-resistant hospital- and community-acquired MRSA (USA300) isolates and antibiotic-sensitive isolates all exhibited similar sensitivities to DIBI as well as to deferiprone (**Table 2**). In contrast to DIBI, deferiprone was only weakly inhibitory for all of the isolates with MICs ranging from 20 to 80 μg/mL, i.e., 0.14–0.58 mM deferiprone.

**Table 2:** *Staphylococcus aureus* isolates tested and their sensitivities to Deferiprone and DIBI.

Clinical isolate	Infection source	Antibiotic resistance	MIC (24h)^∗^			
			**Deferiprone**		**DIBI**	
			**μg/mL**	**μM Fe capacity (3DFP-Fe)^∗∗^**	**μg/mL**	**μM Fe capacity (DIBI-3Fe)^∗∗^**

ATCC43300	Human	MET, GEN, TOB	40	97	2	0.67
ATCCBAA1556	Human Abscess	MRSA USA 300	40	97	4	1.3
WBG525	Human Wound	MET,GEN, TOB, TET	40	97	4	1.3
14-268-06583	Human	MRSA	40	97	2	0.67
ATCC6538	Human Wound	Non-reported	40	97	4	1.3
38	Human Keratitis	Non-reported	40	97	4	1.3
ATCC25923	Human	Non-reported	20	47	1	0.33
12-334-07086	Human Bacteremia	Non-reported	20	47	2	0.67
44L	Canine otitis	ERY	20	47	2	0.67
147	Canine otitis	PEN, CLA	20	47	2	0.67
48	Canine otitis	CLA	20	47	2	0.67
44dx	Canine otitis	Non-reported	20	47	2	0.67
45R	Canine otitis	Non-reported	80	193	2	0.67
10812464	Bovine mastitis	PEN, TET	20	97	4	1.3
22301710	Bovine mastitis	ERY, OXAC,PEN	20	47	2	0.67
32308655	Bovine mastitis	ERY	20	47	2	0.67
21900341	Bovine mastitis	Non-reported	20	47	4	1.3
32101164	Bovine mastitis	Non-reported	20	47	4	1.3

**FIGURE 1:**
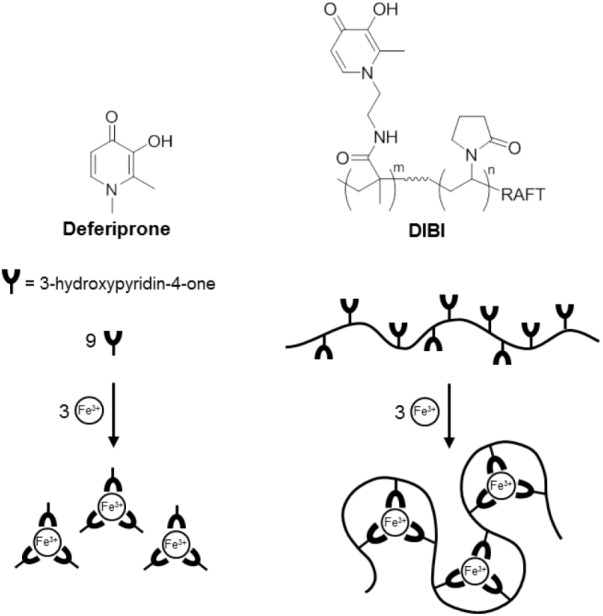
Structure and schematic of Fe (III) binding by Deferiprone and DIBI.

*^∗^MIC, Minimum Inhibitory Concentration at 24h incubation; ^∗∗^, Fe capacity for MIC assuming hexadentate coordination with Chelator-Fe ratio shown (see **Figure 1**); MET, methicillin; GEN, gentamicin; TOB, tobramycin; TET, tetracycline; ERY, erythromycin; PEN, penicillin; CLA, clavulanic acid; OXAC, oxacillin; MET, TOB and OXAC are narrow spectrum and GEN, TET, ERY and PEN are broad spectrum antimicrobials. USA300 is a strain of community-associated methicillin-resistant *Staphylococcus aureus**.

The differences between DIBI and deferiprone MICs remained very large when compared more strictly on their molar iron binding capacities (**Table 2**). Both chelators are hydroxypyridinone (HPO) -containing molecules but differ in overall structure and resulting Fe binding stoichiometries as can be seen in **Figure 1**. For deferiprone, three deferiprone molecules cooperate as chelating ligands to chelate one atom of Fe with full hexadentate coordination. DIBI has a higher MW (9 kDa) and contains 9 HPO residues per molecule all of which have been shown available to bind Fe (**Figure 1** and Ang et al., 2018). Thus, a molecule of DIBI can bind three Fe molecules with full hexadentate binding while three deferiprone molecules share in the binding of one Fe. When compared as to their molar Fe binding capacities as shown in **Table 2**, DIBI still exhibited approximately 100-fold higher inhibitory activity than deferiprone (the difference range for the isolates was 36 to 288-fold).

### DIBI Has Fe-Reversible Activity *in vitro*

**FIGURE 2:**
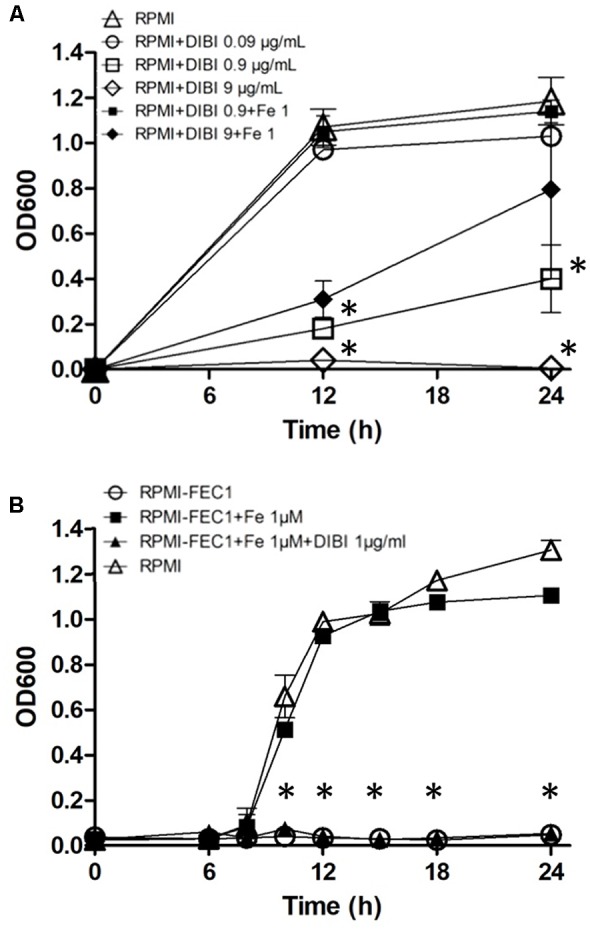
Growth inhibition by DIBI and reversibility by Fe. *Staphylococcus aureus* ATCC43300 was cultured **(A)** in RPMI medium (Δ), RPMI+0.09 μg/mL DIBI (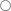), RPMI+0.9 μg/mL DIBI (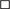), RPMI+9 μg/mL DIBI (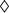), RPMI+0.9 μg/mL DIBI+1 μM iron citrate (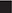) and RPMI+9 μg/mL DIBI+1 μM iron citrate (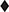) and **(B)** in RPMI-FEC1 medium (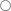), RPMI-FEC1+1 μM iron citrate (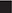), RPMI-FEC1+1 μM iron citrate +0.9 μg/mL DIBI (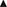) and RPMI medium as control (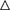). Cultures were shaken continuously at 35°C and growth was measured periodically by measuring OD at 600 nm. Data points reported as means ± SEM from at least two independent experiments. (^∗^) *p* < 0.05 for **(A)** RPMI+0.9 μg/mL DIBI (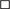) and RPMI+9 μg/mL DIBI (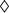) vs. RPMI medium (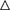) at 12 and 24 h PI and **(B)** RPMI-FEC1+1 μM iron citrate +0.9 μg/mL DIBI (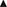) vs. RPMI-FEC1+1 μM iron citrate (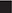) at 10, 12, 15, 18, and 24h PI.

DIBI suppressed growth of *S. aureus* ATCC43300 in a dose-dependent manner (**Figure 2A**). At 0.09 μg/mL (0.01 μM) DIBI showed only a modest effect on growth with a slightly reduced Ymax compared to controls. Additions of 0.9 μg/mL or 9 μg/mL showed increased inhibition with fully suppressed growth observed at 9 μg/mL DIBI (**Figure 2A**). This complete inhibition of growth persisted for at least 36 h (results not shown). Addition of 1 μM supplemental Fe completely reversed the inhibition for cultures containing 0.9 μg/mL DIBI and it provided partial reversal in the case of DIBI at 9 μg/mL. The iron specificity of DIBI inhibition was further examined with cultures grown in RPMI-FEC1. This medium had a very low residual Fe content (0.08 μM) and this was insufficient for any growth measurable by OD600 nm. Fe addition to the RPMI-FEC1 provided a growth rate similar to RPMI control (**Figure 2B**), although the Ymax was slightly lower at 24 h. Addition of DIBI to the Fe-supplemented RPMI-FEC1 cultures also significantly suppressed growth.

### DIBI Suppresses MRSA Wound Infection

**FIGURE 3:**
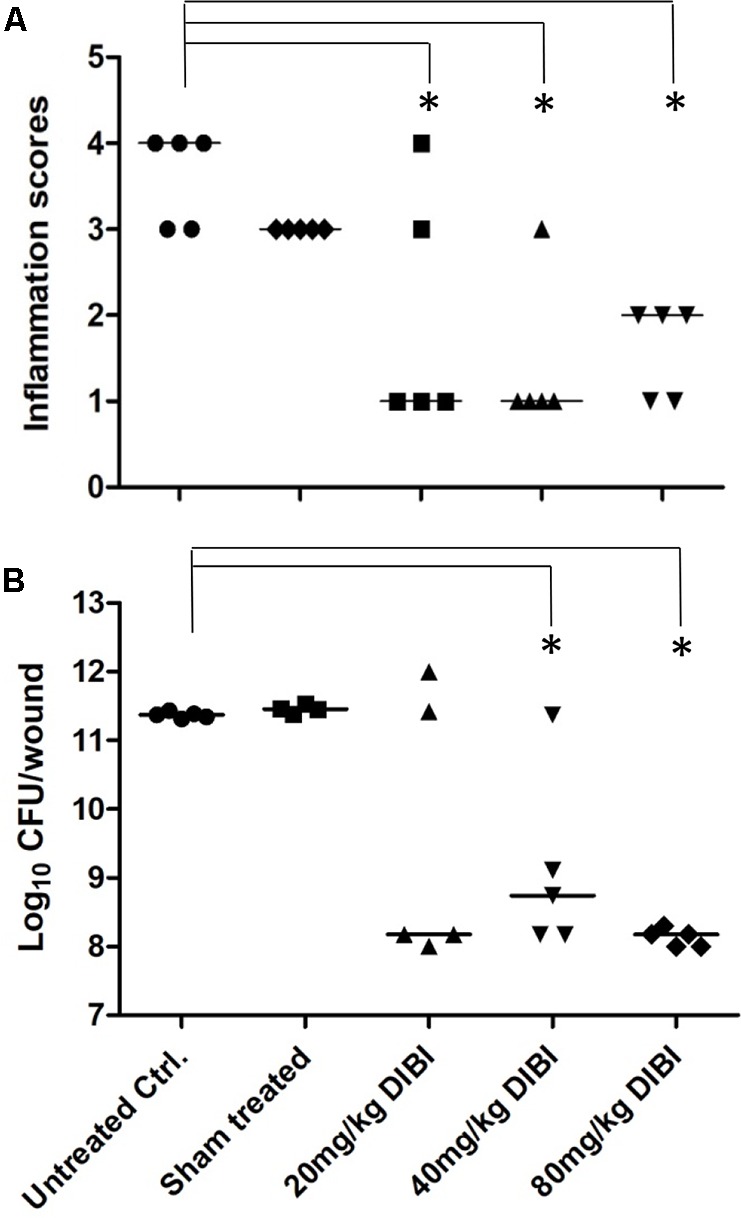
DIBI suppresses *S. aureus* wound infection. Mouse dorsal stripped skin wound patches were infected with *S. aureus* ATCC43300. DIBI was applied topically to the wounds at various dosages. At 4d PI, wounds were scored for inflammation **(A)** and the mice were sacrificed for total wound bacterial CFU burden determination by plate counting **(B)**. Anti-infective responses were evaluated by comparison to the untreated infected controls. Inflammation scores and the log_10_ of bacterial counts (CFU/wound) are presented with their median value. (^∗^) significant with *p* < 0.05.

**FIGURE 4:**
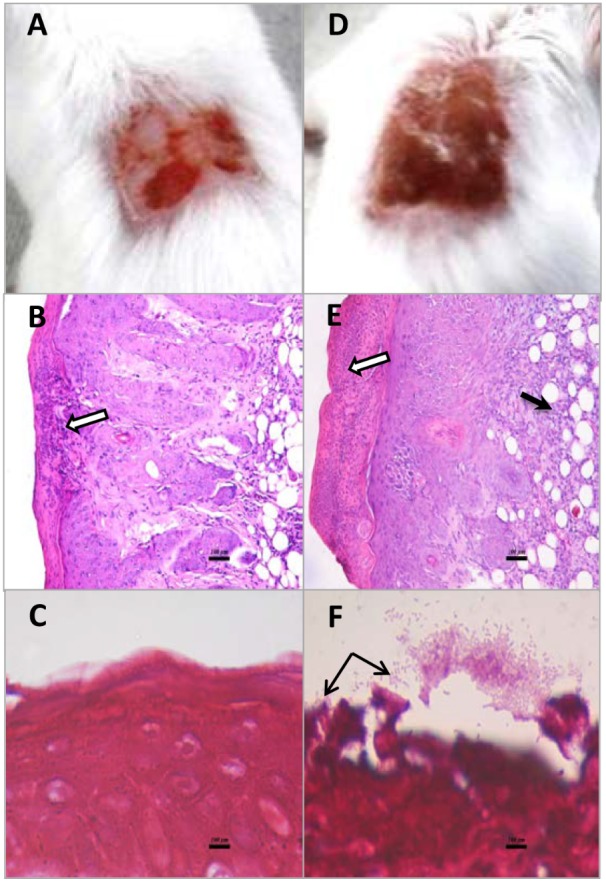
Macroscopic and histopathological appearance of wounds from non-infected mice and mice infected with *S. aureus* ATCC43300 (MRSA). Wounds of non-infected and infected sham-treated mice were observed and photographed at 4d PI **(A,D)**. Tissue samples were removed, fixed, embedded sectioned, and mounted. Sections were stained with hematoxylin/eosin to observe tissue structure **(B,E)** or with crystal violet **(C,F)** to observe bacterial cells. Hypodermal necrosis (white arrows) was infrequent and only focal in nature in control non-infected mice **(B)** but was always present and generally extensive in infected mice **(E)**. Infected mice also exhibited infiltration of mononuclear cells (black arrow in **E**). CV staining showed bacterial cells (black arrows) in and around the necrotic epidermis only in infected mice **(F)**.

Superficial skin wounds infected with *S. aureus* ATCC43300 exhibited a pronounced infection with a 3 log increase in wound bacterial burden over the 4d study period (**Figure 3**). The results for the untreated infection were similar to those reported previously for this model (Kugelberg et al., 2005). Infection was localized to the wound site with only a mild bacteremia (<100 CFU/mL blood) found at 4d PI necropsy and the mice exhibited normal weight gains over the 4 day study period (data not shown). There was a clinically observed inflammation at the wound site that increased over the 4d study period with infected mice showing inflammation scores of approximately 3.5 out of a maximum 4 (**Figure 3A**). DIBI applied topically to the infected wounds resulted in a dose-dependent suppression of infection as evidenced both by a reduced total bacterial wound burden and a reduced inflammation score when compared to the untreated control (**Figure 3**). Sham-treated infected controls that were treated with only the vehicle, as was used for DIBI application, exhibited a similar course of infection to that of untreated infected controls. Histological examination of wounded non-infected and wounded infected sham-treated mice provided useful information as to the nature of the infection and its pathology. For wounded but non infected control mice, three of the four mice examined had no evident pathological abnormalities in H&E stained tissue sections. The one mouse that displayed wound abnormalities had no overt signs of infection (**Figure 4A**) but H&E stained tissue sections displayed multifocal epidermal necrosis with minimal neutrophilic infiltration in the hypodermis as shown in **Figure 4B**. CV stained sections from this mouse showed no bacteria within the wound (**Figure 4C**). These results were consistent with wounded non-infected mice showing relatively fast and normal repair of their wounds over the 4 day period. In contrast, all four of the infected sham-treated mice exhibited overt signs of infection (**Figure 4D**) with wound pathology in the form of moderate to severe epidermal necrosis and with focal to diffuse edema and marked neutrophilic infiltration as observed in H&E stained sections (**Figure 4E**). CV stained sections from these infected mice showed bacterial presence within the wound, i.e., around the epidermis (**Figure 4F**).

### DIBI Suppresses Nares MRSA Carriage

**FIGURE 5:**
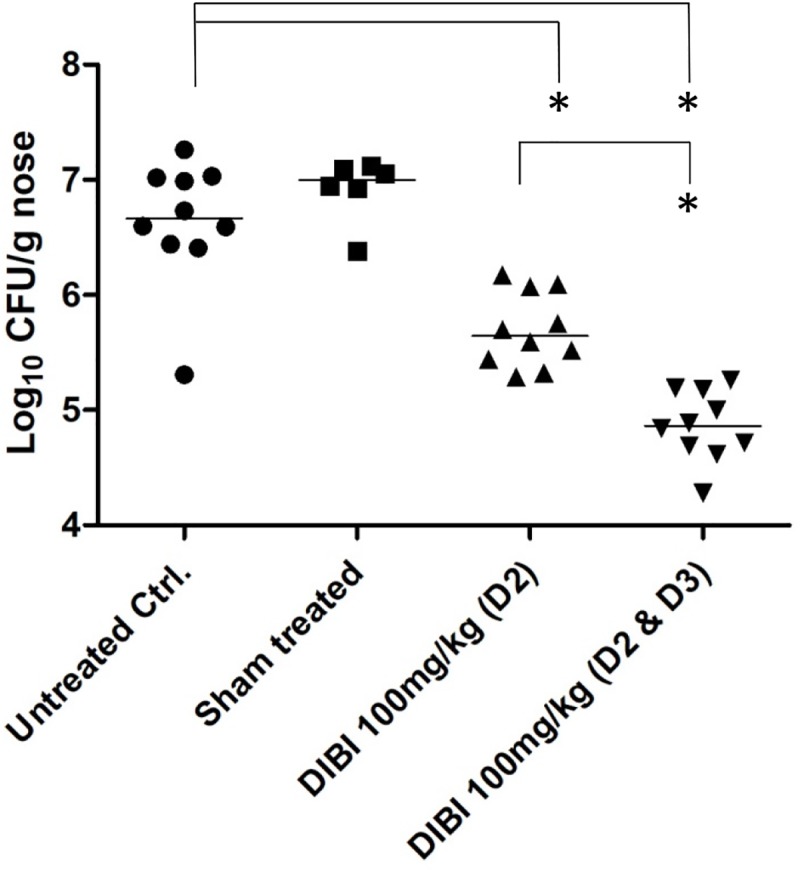
DIBI reduces *S. aureus* nares carriage. Mice were infected intranasally with *S. aureus* ATCC43300 at day -2 and day 0. At day 2 PI mice were treated intranasally with PBS-vehicle or with DIBI at 100 mg/kg. Groups of mice that had been treated with DIBI at day 2 PI were also treated at day 3 PI. Mice were sacrificed at day 5 PI and quantitative determinations of CFU/g nares were completed by plate counting. The log_10_ of bacterial counts (CFU/g nares) are graphed with their median value. Carriage burden of treatment groups were compared to non-treated infected controls; (^∗^) significant with *p* < 0.05.

DIBI was also assessed for its effects on MRSA nares carriage burden following infection of mice nares with *S*. *aureus* ATCC43300. Bacterial introduction by intra-nasal (IN) instillation resulted in a sustained carriage of *S*. *aureus* at 5 days PI and with a total nose bacterial burden of 6.6 log_10_CFU in control infected mice (**Figure 5**). The control bacterial burdens were similar to those previously reported for this nares colonization model (Chhibber et al., 2014). There were no overt clinical signs of infection over the 6 day study period. *S. aureus* colonies or other resident flora were not recovered from control non-infected mice (results not shown).

Intranasal administration of DIBI (100 mg/kg equally divided to each nostril) on day 2 PI resulted in a >1 log reduction (*p* < 0.05) in the nares bacterial burden by day 5 PI but sham treatment using only IN instillation of the PBS vehicle had no effect on the bacterial burden (**Figure 5**). DIBI administration on both day 2 and day 2 + 3 PI resulted in a >2 log reduction in bacterial burden by day 5 PI.

### DIBI Does Not Impair but Enhances Antibiotic Activities

The possible effects of DIBI on the activities of various antibiotics representative of discrete chemical classes (aminoglycosides, fluoroquinolones, and glycopeptides) that are typically used clinically for staphylococcal infections in humans or animals were examined. The main objectives were to determine first if DIBI might affect antibiotic killing and secondly if DIBI might affect recovery growth of bacterial survivors following a sub-lethal exposure to an antibiotic. Reference strain ATCC6538 which is often employed to assess new antimicrobials was studied. Preliminary checkerboard assays revealed FICI values of less than 0.5 suggesting synergy of DIBI with each of the antibiotics. Calculated FICIs were 0.04 for GEN, 0.04 for CIP, and 0.13 for VAN. However, we observed a growth trailing effect with DIBI when present with the antibiotics in checkerboards which made visual reading of growth/no-growth endpoints somewhat difficult. Given these limitations and the need to assess DIBI responses in a dynamic assay, we focussed on a time kill/recovery growth assay system. The initial inoculum of approximately 10^5^ CFU/mL provided sufficiently large bacterial numbers to assess killing but yet a low enough initial inoculum to require significant replicative growth in order to reach Ymax CFU/mL values.

**FIGURE 6:**
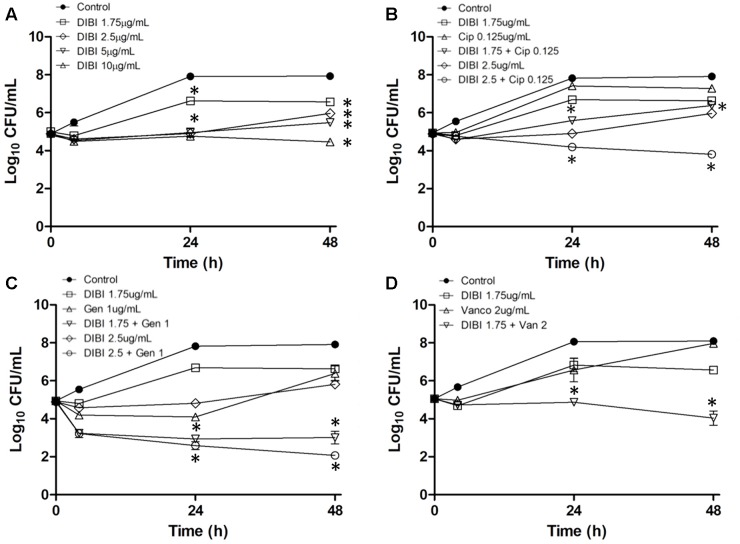
Influence of DIBI on antibiotic killing and recovery growth. *S. aureus* ATCC6538 was inoculated at a final concentration of approximately 10^5^ CFU/mL into RPMI or RPMI containing CIP, GEN, VAN, or DIBI either alone or combined with these antibiotics. Cultures were grown at 35–37°C with bacterial counts (CFU/mL) being determined at intervals over 48 h. **A:** Control (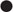); DIBI added at 1.75 (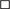); 2.5 (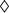); 5 (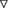) or 10 μg/mL (Δ). **B**: Control (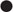); DIBI 1.75 μg/mL (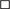); DIBI 2.5 μg/mL (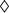); CIP 0.125 μg/mL (Δ); DIBI 1.75 μg/mL+CIP 0.125 μg/mL (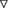) and DIBI 2.5 μg/mL+CIP 0.125 μg/mL (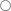). **C**: Control (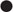); DIBI 1.75 μg/mL (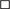); DIBI 2.5 μg/mL (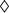); GEN 1 μg/mL (Δ); DIBI 1.75 μg/mL+GEN 1 μg/mL (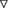) and DIBI 2.5 μg/mL+GEN 1 μg/mL (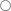). **D**: Control (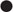); DIBI 1.75 μg/mL (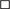); VAN 2 μg/mL (Δ) and DIBI 1.75 μg/mL+VAN 2 μg/mL (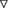). Data points are reported as means ± SEM. (^∗^) *p* < 0.05 for DIBI+antimicrobial combinations vs. DIBI and antimicrobial treatments alone.

A concentration of DIBI providing partial inhibition of growth on its own was determined first (**Figure 6A**). DIBI added at 1 μg/mL had no apparent effect on growth while concentrations ≥1.75 μg/mL reduced overall growth in a dose-dependent manner. A DIBI concentration of 1.75 μg/mL provided an Fe-chelating capacity of approximately 0.6 μM only in slight excess of the average Fe content of RPMI (see **Table 1**). A DIBI concentration of 10 μg/ml appeared to provide slight bactericidal activity (**Figure 6A**). Based on these results a DIBI concentration range of 1.75–2.5 μg/mL was selected on the basis that it would result in slowed but continued growth, i.e., typical of iron-restricted growth physiology.

Similarly, CIP, GEN, and VAN concentrations were determined that provided some initial killing on their own, followed by significant recovery growth by 24–48 h post exposure (PE) (see **Figures 6B–D**). Kill/growth responses were then compared for antibiotic alone, DIBI alone and for the DIBI/antibiotic combinations all as compared to untreated control growth.

CIP at a ½ MIC (0.125 μg/mL) provided short term killing followed by strong recovery growth after 4 h PE with bacterial numbers approaching those of controls by 24 h (**Figure 6B**). DIBI at 1.75 μg/mL or 2.5 μg/mL together with CIP did not affect initial killing by CIP. However, in the case of CIP with 2.5 μg/mL DIBI, a continuing slow rate of killing was observed to 24 h PE and this continued to 48 h PE. DIBI at 1.75 μg/mL with CIP resulted in a substantially reduced recovery growth after the initial killing (**Figure 6B**).

ATCC6538 treated with a ½ MIC (1 μg/mL) GEN exhibited approximately a 1 log initial kill over the first 4 h followed with little change in CFU/ml to 24 h PE but then followed by >2 log CFU/ml recovery growth between 24 and 48 h PE (**Figure 6C**). DIBI at either 1.75 or 2.5 μg/mL along with GEN resulted in an increased kill rate and overall extent of killing with little recovery growth occurring after 24 h PE in the case of the 1.75 μg/mL DIBI/GEN combination. At the higher DIBI concentration of 2.5 μg/mL with GEN there was further killing to 48 h PE (**Figure 6C**). There was approximately a 4 log CFU/mL difference with the DIBI/GEN combination compared to either the DIBI alone or GEN alone treatments at 48 h PE.

VAN at 1 MIC (2 μg/mL) provided only slight killing of ATCC6538 followed by strong recovery growth by 24 and 48 h PE (**Figure 6D**). DIBI at 1.75 μg/mL with VAN provided slightly enhanced killing and fully suppressed recovery growth to 24 h PE and also to 48 h PE with evidence of slight additional killing occurring between 24 and 48 h PE (**Figure 6D**).

## Discussion

DIBI, an iron chelating polymer was found to be strongly inhibitory to a diverse group of *S. aureus* isolates irrespective of their animal source of origin (human, cattle or dogs) and irrespective of their antibiotic resistance characteristics. Both hospital- and community-acquired (USA300) MRSA had similar sensitivities.

DIBI also suppressed *S. aureus* infection *in vivo* when applied topically to infected superficial skin wounds or by intranasal administration to MRSA-colonized nares. A DIBI dose-dependent reduction in bacterial burdens and a reduction in wound infection-associated inflammation were observed. This evidence was consistent with DIBI sequestering host Fe within wounds or on the mucosal surfaces of the nares, i.e., host Fe supplies that would otherwise have been available to support *S. aureus* infection. The *in vivo* results are especially significant given the recent demonstration of *S. aureus* upregulating one of its key iron acquisition systems during experimental infection (Bacconi et al., 2017) revealing the bacterium is forced to compete continuously for host Fe during the course of infection. The previous finding that various Fe acquisition genes are upregulated in *S. aureus* while colonizing the nares (Chaves-Moreno et al., 2016) provides further evidence that host Fe availability is important for staphylococcal growth *in vivo* and that effective sequestration of host iron might suppress nares colonization and other infections. Our results provide evidence that the innate host iron sequestration defense mechanisms can be augmented by DIBI to better control infection.

An iron chelator such as DIBI offers potential broad utility as an alternative anti-infective for treating staphylococcal infections in man and other animals. Our ongoing investigations with canine otitis externa have shown that DIBI has stand-alone potential as a “non-antibiotic” anti-infective for treating otitis caused by *Malassezia pachydermatis* and staphylococci and also for other Gram+ and Gram– bacterial infections (unpublished results).

DIBI is a linear hydroxypyridinone-containing polymer of relatively low molecular weight (9 kDa) and it was found to have potent activity in contrast to deferiprone. Deferiprone is a small hydroxypyridinone molecule (139 Da) and a close chemical relative of DIBI with both chelators possessing identical 3-hydroxypyridin-4(1H)-one iron binding moieties. Compared on their molar iron binding capacities, DIBI was approximately 100-fold more inhibitory for *S. aureus* than deferiprone (full range of fold difference between 36 and 288-fold). This finding suggests that DIBI chelates Fe with a higher avidity than deferiprone and/or the corresponding DIBI-sequestered Fe is less available to *S. aureus* than is deferiprone-sequestered Fe. Given the larger molecular size of DIBI, it seems unlikely it would be freely transportable for intracellular uptake by microorganisms while deferiprone would presumably be available intracellularly. We have also reported enhanced growth inhibition by DIBI over deferiprone for *Candida albicans* (Savage et al., 2018) and *Acinetobacter baumannii* (Ang et al., 2018) as well as for breast cancer cells (Power Coombs et al., 2015).

Chemical characterization studies (Ang et al., 2018) have revealed that DIBI’s hydroxypyridinone groups are widely distributed on its polyvinylpyrrolidone backbone. The resulting platform appears to provide enhanced iron chelating properties consistent with the DIBI molecule wrapping around its chelated Fe, this possibly providing some shielding of its chelated Fe atoms within the overall structure as depicted in **Figure 1**.

DIBI’s inhibitory activity was also shown to be Fe-specific and the addition of iron citrate reversed this inhibition. FEC1 treatment of the culture media was useful for investigating this aspect. FEC1 is a Fe-chelating adsorbent with identical iron chelating functionality to DIBI except that it is in the form of a crosslinked polymeric structure which is insoluble in water. FEC1 therefore provides a tool for treating a growth medium followed by its physical removal with chelated Fe (Holbein and Mira de Orduña, 2010). FEC1 removed primarily Fe and Mn from MHB and RPMI without affecting other major essential cations such as Ca and Mg. This aspect of metal selectivity has importance as previous studies of *S. aureus* Fe requirements employing deferrated media had utilized Chelex resin to extract Fe (Courcol et al., 1991), but this resin contains iminodiacetic acid metal binding groups similar to EDTA and these are much less metal selective. Consequently, Chelex^®^ removes a variety of other cations including Ca and Mg. The lack of Fe selectivity of EDTA precludes its use as an anti-infective and also makes conclusions as to microbial metal importance using EDTA or Chelex questionable as tools.

Mn removal by FEC1 was expected and this was consistent with the known atomic similarities between Fe and Mn, as evidenced, for example, with their similar metal reactive centers in superoxide dismutases (Miller, 2004). Mn removal from our growth media could have significance given its reported importance for *S. aureus* (Cassat and Skaar, 2012). However, we have shown that normal growth kinetics and Ymax growth yield were restored upon re-addition of only Fe to RPMI-FEC1 while deferrated RPMI-FEC1 medium without re-added Fe provided no apparent growth. Our results were consistent with DIBI being Fe-specific as well as Fe having a principal role for staphylococcal growth.

An Fe-chelating agent such as DIBI would be expected to broadly affect synthesis and regulation of a wide array of Fe-dependent microbial enzymes (e.g., ribonucleotide reductase, citrate aconitase, cytochromes, catalase, superoxide dismutase, etc) and thus, exert a generalized physiological stress on microbes. Thus, it became important to investigate DIBI’s potential interaction with antibiotics given that DIBI might be administered in conjunction with these agents. A key question addressed was whether DIBI might affect the killing activity of different antibiotics. We studied three antibiotics including: GEN, an aminoglycoside class protein synthesis inhibitor; CIP a fluoroquinolone class DNA synthesis inhibitor and VAN a glycopeptide class cell wall synthesis inhibitor. Each of these antibiotics or other members of their respective families are currently used to treat staphylococcal infections in either humans or animals. Antibiotics were tested in our studies at a concentration that provided only partial initial killing followed by strong recovery growth. Antibiotic therapy typically employs a high dosage (i.e., multiple of the MIC) to ensure maximal killing but the condition of low sub-MIC antibiotic exposure with a survivor population does have clinical relevance representing a condition favoring potential microbial re-growth/infection and also a condition of possible positive selection for antibiotic resistant survivors.

For our DIBI/antibiotic interaction studies, we also deliberately provided only a sub-inhibitory amount of DIBI so as to ensure a physiological condition of Fe-restricted growth, i.e., growth restricted but not fully arrested. This physiological state of Fe-restricted growth could also have clinical relevance to infection noting that *S. aureus* has been shown to respond to *in vivo* Fe restriction through upregulation of its Fe acquisition mechanisms throughout the entire course of infection (Chaves-Moreno et al., 2016; Bacconi et al., 2017).

A small amount of DIBI (1.75–2.5 μg/mL) that was only in mild excess of that required to theoretically chelate the free Fe content of the test medium resulted in only a slight killing of the initial bacterial population followed by slow and continued growth over the 48 h study period. Fe-restricted growth resulting from this sub-MIC DIBI enhanced the overall activities of GEN, CIP, and VAN. We also observed similar responses with ATCC43300 (results not shown).

Several aspects of the observed enhancing activities of DIBI with these antibiotics are noteworthy. Importantly, DIBI did not appear to impair the initial killing activity of any of the antibiotics tested. DIBI actually increased or extended the initial antibiotic killing phase to varying degrees for each antibiotic. DIBI also appeared to either strongly restrict or fully block recovery growth that was typically observed with a sub-MIC of antibiotic alone, i.e., re-growth following the initial killing phase from the antibiotic. The DIBI/antibiotic responses for each antibiotic thus appeared to be synergistic in nature and not just additive. DIBI promoted additional and prolonged killing by the antibiotics especially for GEN.

Our results showing DIBI enhancement of VAN activity were in general agreement with the previous report that the iron chelator deferasirox enhanced VAN activity against MRSA (Luo et al., 2014). However, their reported enhancement *in vitro* required 50 μg/mL deferasirox corresponding to 70 μM Fe binding capacity (noting two molecules of deferasirox coordinate one Fe atom). Our VAN enhancement by DIBI required only 1.75 μg/mL DIBI corresponding to only 0.52 μM Fe binding capacity. Thus, DIBI was >125 times more active to enhance VAN than had been reported for deferasirox. Deferiprone has also been studied as to its potential antibiotic enhancing activities against *S. aureus* (Richter et al., 2017). Deferiprone alone at 20 mM (2.8 mg/mL) did not enhance CIP or GEN anti-biofilm activity in an artificial wound model but when present with gallium-protoporphyrin it did enhance CIP activity. The Ga-heme, being a nonfunctional analog for Fe-heme, in *S. aureus*, appeared to have exerted additional Fe requirement stress on the bacteria (Richter et al., 2017). The earlier demonstration that the host defense iron sequestering protein lactoferrin enhances penicillin G activity for *S. aureus* isolated from bovine mastitis (Diarra et al., 2002) provides additional evidence that Fe limitation has potential for improving antibiotic responses.

It seems reasonable that repair of antibiotic-effected damage might first be required so as to then enable subsequent regrowth and replication, and these repair mechanisms would likely include Fe-dependent enzymes. Restriction of microbial essential Fe by DIBI might then generally affect this essential bacterial repair for recovery/regrowth. Interestingly, the three antibiotics studied have widely different bacterial targets and modes of action. The present results and others showing that DIBI enhances clearance of *Candida albicans* infection by fluconazole (Savage et al., 2018) suggest DIBI enhancement of antimicrobial activity might be broad-based with potential for improving antibiotic responses for various microbial infections. Our ongoing studies are investigating the effects of DIBI *in vivo* both on its own and in combination with antibiotics for anti-infective therapy of both Gram positive and Gram negative bacterial infections.

## Author Contributions

All authors listed have made a substantial, direct and intellectual contribution to the work, and approved it for publication.

## Conflict of Interest Statement

BH has a beneficial interest in Chelation Partners Inc., that has contributed financially to this study. MdCP, KS, and DA are employees of Chelation Partners Inc., RD has no conflicts or competing interests in relation to this study.
